# Lipid-Based Antimicrobial Delivery-Systems for the Treatment of Bacterial Infections

**DOI:** 10.3389/fchem.2019.00872

**Published:** 2020-01-10

**Authors:** Da-Yuan Wang, Henny C. van der Mei, Yijin Ren, Henk J. Busscher, Linqi Shi

**Affiliations:** ^1^State Key Laboratory of Medicinal Chemical Biology, Key Laboratory of Functional Polymer Materials, Ministry of Education, Institute of Polymer Chemistry, College of Chemistry, Nankai University, Tianjin, China; ^2^Department of Biomedical Engineering, University of Groningen and University Medical Center Groningen, Groningen, Netherlands; ^3^Department of Orthodontics, University of Groningen and University Medical Center Groningen, Groningen, Netherlands

**Keywords:** bacterial biofilm, micelles, zeta potentials, hydrophobicity, lipids, liposomes, infection, fusogenicity

## Abstract

Many nanotechnology-based antimicrobials and antimicrobial-delivery-systems have been developed over the past decades with the aim to provide alternatives to antibiotic treatment of infectious-biofilms across the human body. Antimicrobials can be loaded into nanocarriers to protect them against de-activation, and to reduce their toxicity and potential, harmful side-effects. Moreover, antimicrobial nanocarriers such as micelles, can be equipped with stealth and pH-responsive features that allow self-targeting and accumulation in infectious-biofilms at high concentrations. Micellar and liposomal nanocarriers differ in hydrophilicity of their outer-surface and inner-core. Micelles are self-assembled, spherical core-shell structures composed of single layers of surfactants, with hydrophilic head-groups and hydrophobic tail-groups pointing to the micellar core. Liposomes are composed of lipids, self-assembled into bilayers. The hydrophilic head of the lipids determines the surface properties of liposomes, while the hydrophobic tail, internal to the bilayer, determines the fluidity of liposomal-membranes. Therefore, whereas micelles can only be loaded with hydrophobic antimicrobials, hydrophilic antimicrobials can be encapsulated in the hydrophilic, aqueous core of liposomes and hydrophobic or amphiphilic antimicrobials can be inserted in the phospholipid bilayer. Nanotechnology-derived liposomes can be prepared with diameters <100–200 nm, required to prevent reticulo-endothelial rejection and allow penetration into infectious-biofilms. However, surface-functionalization of liposomes is considerably more difficult than of micelles, which explains while self-targeting, pH-responsive liposomes that find their way through the blood circulation toward infectious-biofilms are still challenging to prepare. Equally, development of liposomes that penetrate over the entire thickness of biofilms to provide deep killing of biofilm inhabitants still provides a challenge. The liposomal phospholipid bilayer easily fuses with bacterial cell membranes to release high antimicrobial-doses directly inside bacteria. Arguably, protection against de-activation of antibiotics in liposomal nanocarriers and their fusogenicity constitute the biggest advantage of liposomal antimicrobial carriers over antimicrobials free in solution. Many Gram-negative and Gram-positive bacterial strains, resistant to specific antibiotics, have been demonstrated to be susceptible to these antibiotics when encapsulated in liposomal nanocarriers. Recently, also progress has been made concerning large-scale production and long-term storage of liposomes. Therewith, the remaining challenges to develop self-targeting liposomes that penetrate, accumulate and kill deeply in infectious-biofilms remain worthwhile to pursue.

## Introduction

The threat posed to mankind of hard to treat, antibiotic-resistant infectious biofilms is better realized world-wide than ever. With cancer being considered more and more as a chronic disease, infection by antibiotic-resistant bacteria is expected to become the number one cause of death by the year 2050 (Humphreys and Fleck, 2016). This frightening scenario has many reasons. First of all, infectious biofilms are tenacious by nature and antimicrobials have difficulty penetrating the biofilm matrix embedding its bacterial inhabitants (Gupta et al., 2018). The biofilm matrix is composed of Extracellular Polymeric Substances (EPS) (Bjarnsholt et al., 2013) containing proteins, polysaccharides, humic acids, and eDNA (Flemming et al., 2016). The EPS-matrix acts as a glue holding biofilm-bacteria together and protecting them against the host immune system and environmental challenges, amongst which antimicrobials (Liu et al., 2019a). Secondly, rampant overuse of antibiotics has yielded, and still is yielding new antibiotic-resistant strains that cannot be killed by known antibiotics (Neville and Jia, 2019). Thirdly, development of new antibiotics is stalling (N'Guessan et al., 2018; Jangra et al., 2019), because their effective life-time before the first resistant strains arise, is becoming shorter and shorter, decreasing the incentive for commercialization and therewith clinical use of new antibiotics (Liu et al., 2019b).

**Figure 1:**
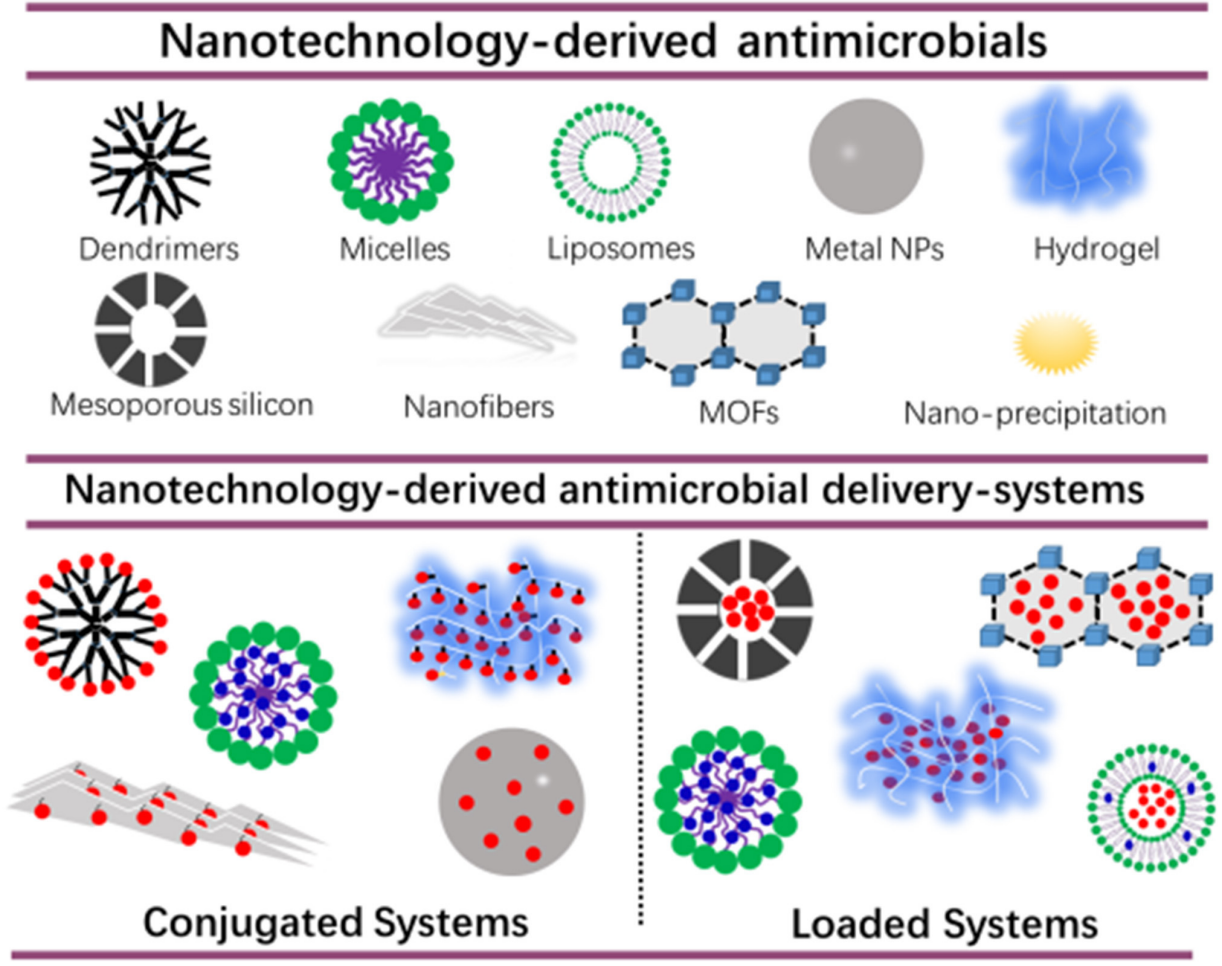
Nanotechnology-derived antimicrobial delivery-systems, including nanofiber-composed hydrogels. Delivery-systems are divided into systems in which antimicrobials are conjugated to a carrier or loaded into a carrier. Hydrophobic and hydrophilic antimicrobials are indicated in blue and red, respectively.

A first challenge in the development of new infection-control strategies, is to develop an antimicrobial or antimicrobial delivery-system that allows the antimicrobial to penetrate deeply into a biofilm and kill biofilm-bacteria across the entire thickness of the biofilm (Drbohlavova et al., 2013; Liu et al., 2019a). Many nanotechnology-based drugs and drug-delivery-systems have been developed over the past decades with the aim of self-targeting, penetrating and eradicating tumors (Kong et al., 2019; Majumder et al., 2019; Paunovska et al., 2019). Biofilms and tumors are on the one hand very different, yet are both characterized by a low pH environment, allowing self-targeting of pH adaptive, smart carriers (Liu et al., 2016). Also, their clinical treatment poses the same challenges, including prevention of resistance and recurrence. Not surprisingly, new strategies for infection-control are arising nowadays, that are derived from technologies initially designed for tumor treatment. Figure 1 gives an overview of nanotechnology-derived antimicrobial delivery-systems currently considered for infection-control, many of which are derived from new tumor treatment strategies.

Nanotechnology-derived antimicrobial delivery systems have excellent biocompatibility, and can be designed to be environmentally-responsive and self-targeting (Lopes and Brandelli, 2018; Wolfmeier et al., 2018; Zhao et al., 2018), provided their diameter is below the limit for reticulo-endothelial rejection of around 100–200 nm (Wang et al., 2019). However, without suitable functionalization of their outermost surface or drug-loading (Figure 1), their antimicrobial efficacy is usually low. In conjugated systems, antibiotics, peptides or other antimicrobials are bound to dendrimers (Kumar et al., 2015; Xue et al., 2015), and hydrogels (Zendehdel et al., 2015) which should be done carefully in order not to sacrifice bio-active groups. To a certain extent, this restricts the application of antimicrobial-conjugated systems. Alternatively, antimicrobials can be loaded into nanotechnology-derived antimicrobial delivery-systems, to protect antimicrobials underway through the blood circulation from de-activation, reduce their toxicity and prevent potential, harmful side-effects of the antimicrobials. Moreover, antimicrobial nanocarriers can be equipped with stealth and pH-responsive features that allow self-targeting and accumulation in infectious biofilms at high concentrations. Micelles can be made for instance, consisting of a hydrophilic poly(ethylene glycol) (PEG)-shell and pH-responsive poly(β-amino ester) (PAE). This renders stealth properties to the micelles at physiological pH due to the exposure of the PEG-shell allowing their presence in the blood circulation without negative side-effects and penetration in a tumor or infectious biofilm. However, once in a more acidic, pathological site, such as in a tumor (Ray et al., 2019) or biofilm (Liu et al., 2016; Wu et al., 2019) (becoming even more acidic toward its bottom; Peeridogaheh et al., 2019), pH-responsive PAE groups become positively-charged causing self-targeting and accumulation (Liu et al., 2012, 2016). Micelles are more suitable for functionalizing of their surface without affecting their hydrophilicity ratio than liposomes, because of the relatively low molecular weight of the lipids involved in liposomes (1,200–1,800 g/mol) compared with the surfactants used in micelles (>8,000 g/mol). Inadvertent leakage remains a concern in antimicrobial-loaded systems (Kim et al., 2019).

The two most common nanocarriers considered for drug loading are micelles and lipid-based liposomes. The structure and composition of liposomes, also known as vesicles, bear similarity to the one of cell membranes. The main difference between micelles and liposomes is the hydrophilicity of their outer surface and inner core (Table 1). Micelles are self-assembled, spherical core-shell structures composed of a single layer of surfactants, with a hydrophilic head-group and a hydrophobic tail-group pointing to the micellar core. Liposomes are composed of lipids and due to their amphiphilic nature can assemble into bilayers, similar to the structure and composition of cell membranes. The hydrophilic head of the lipids determines the surface properties of liposomes, while the hydrophobic tail, internal to the bilayer, determines the fluidity of liposomal membranes. Therefore, whereas micelles can only be loaded with hydrophobic antimicrobials of which there are few candidates, hydrophilic antimicrobials can be encapsulated in the hydrophilic, aqueous core of liposomes and hydrophobic or amphiphilic antimicrobials can be inserted in the phospholipid bilayer. As a consequence, the number of candidate antimicrobials for liposome-loading, is relatively large, while the loading capacity of liposomes is relatively high (Ehsan and Clancy, 2015; Liu et al., 2019a; see also Table 1).

**Table 1:** Main differences between liposomal and micellar drug carriers, candidate antimicrobials for loading into liposomes or micelles and the relative advantages of both types of nanocarriers.

**Liposomes**	**Micelles**
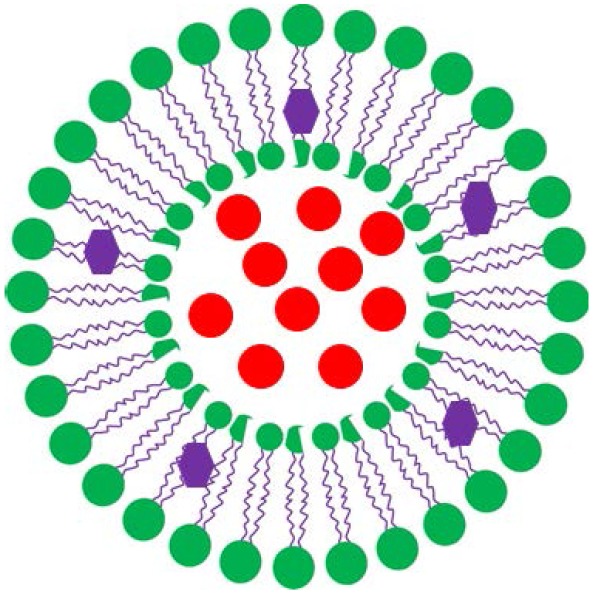	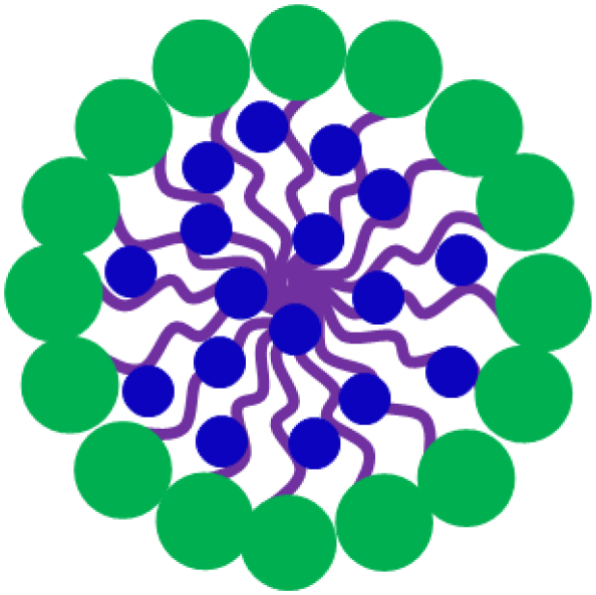
**Candidate antimicrobials for loading**	**Candidate antimicrobials for loading**
Amikacin (Mugabe et al., 2006), Gentamicin (Mugabe et al., 2005, 2006), Tobramycin (Sachetelli et al., 2000; Marier et al., 2002; Mugabe et al., 2006; Messiaen et al., 2013), Triclosan (Sanderson et al., 1996), Vancomycin (Nicolosi et al., 2010; Chakraborty et al., 2012), Azithromycin (Solleti et al., 2015), Metronidazole (Vyas et al., 2001), Oxacillin (Meers et al., 2008), Daptomycin (Hu et al., 2019), Antimicrobial peptides (Dashper et al., 2005)	Triclosan (Liu et al., 2016), Curcumin (Huang et al., 2017), Silver NPs (Lin et al., 2019), Rifampicin and isoniazid (Praphakar et al., 2019), Bedaquiline (Soria-Carrera et al., 2019)
**Liposome advantages**	**Micelle advantages**
- Hydrophilic and hydrophobic antimicrobial loading - High loading capacity - Intra-cellular release of cargo through fusion with bacterial cell membranes - Fusogenicity at the expense of cargo leakage - FDA approved dosage forms for clinical use	- Relatively little leakage of hydrophobic cargo - Relatively easy functionalization

*Hydrophobic and hydrophilic antimicrobials are indicated in blue and red, respectively*.

**Figure 2:**
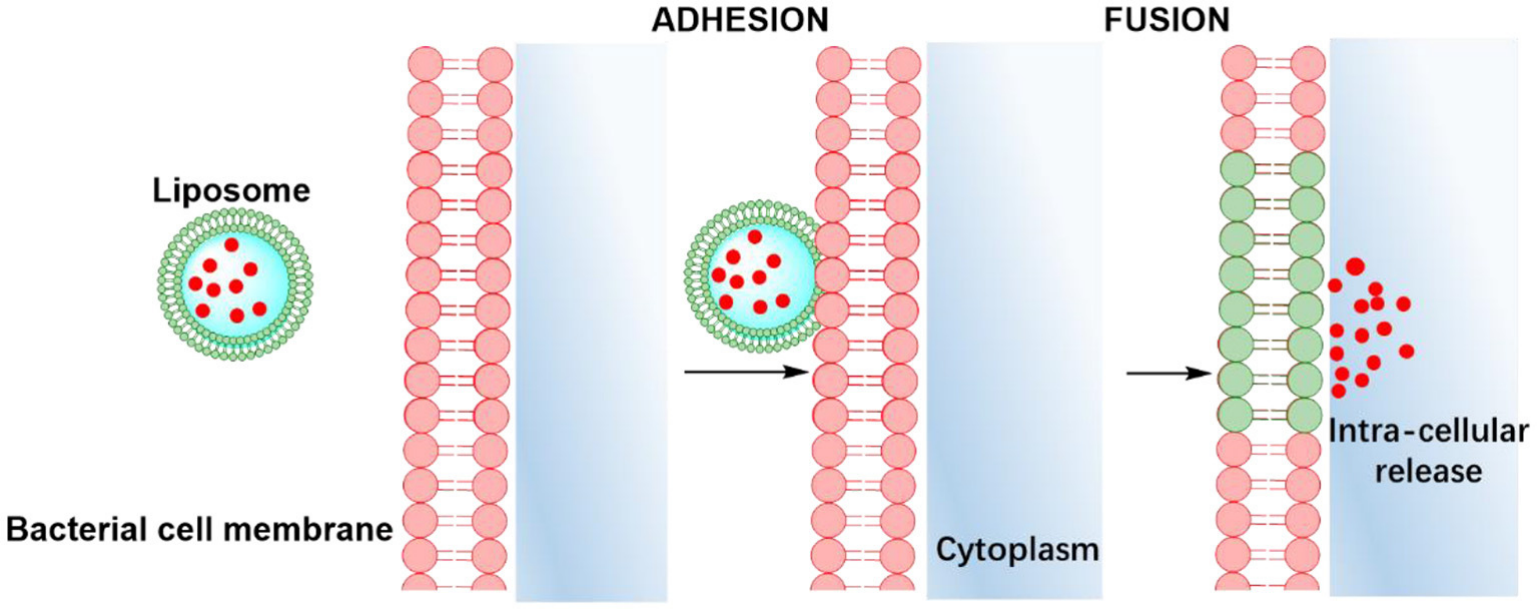
Similarity-mediated fusion of liposomes into bacterial cell membranes and release of antimicrobial cargo into a bacterium.

Apart from offering a wider choice of candidate antimicrobials for loading and higher loading, another advantage of lipid-based antimicrobial delivery-system is their fusogenicity, i.e., the ability of liposomes to fuse with the outer membrane of bacteria (see also Table 1), due to the fluidity of their phospholipid bilayer structure. The liposomal phospholipid bilayer resembles the structure of bacterial cell membranes, which facilitates fusion based on similarity (Figure 2). Upon fusion, high antimicrobial-doses are directly available inside a bacterium (Akbarzadeh et al., 2013).

In this review, we summarize the different types of lipid-based antimicrobial delivery-systems according to their lipid bilayer composition, membrane fluidity, outer surface properties and ability to trigger the release of the encapsulated antimicrobials upon fusion. Applications and perspectives of liposomal, antimicrobial delivery-systems for the treatment of bacterial infections will be discussed.

## Preparation of Liposomes

**Figure 3:**
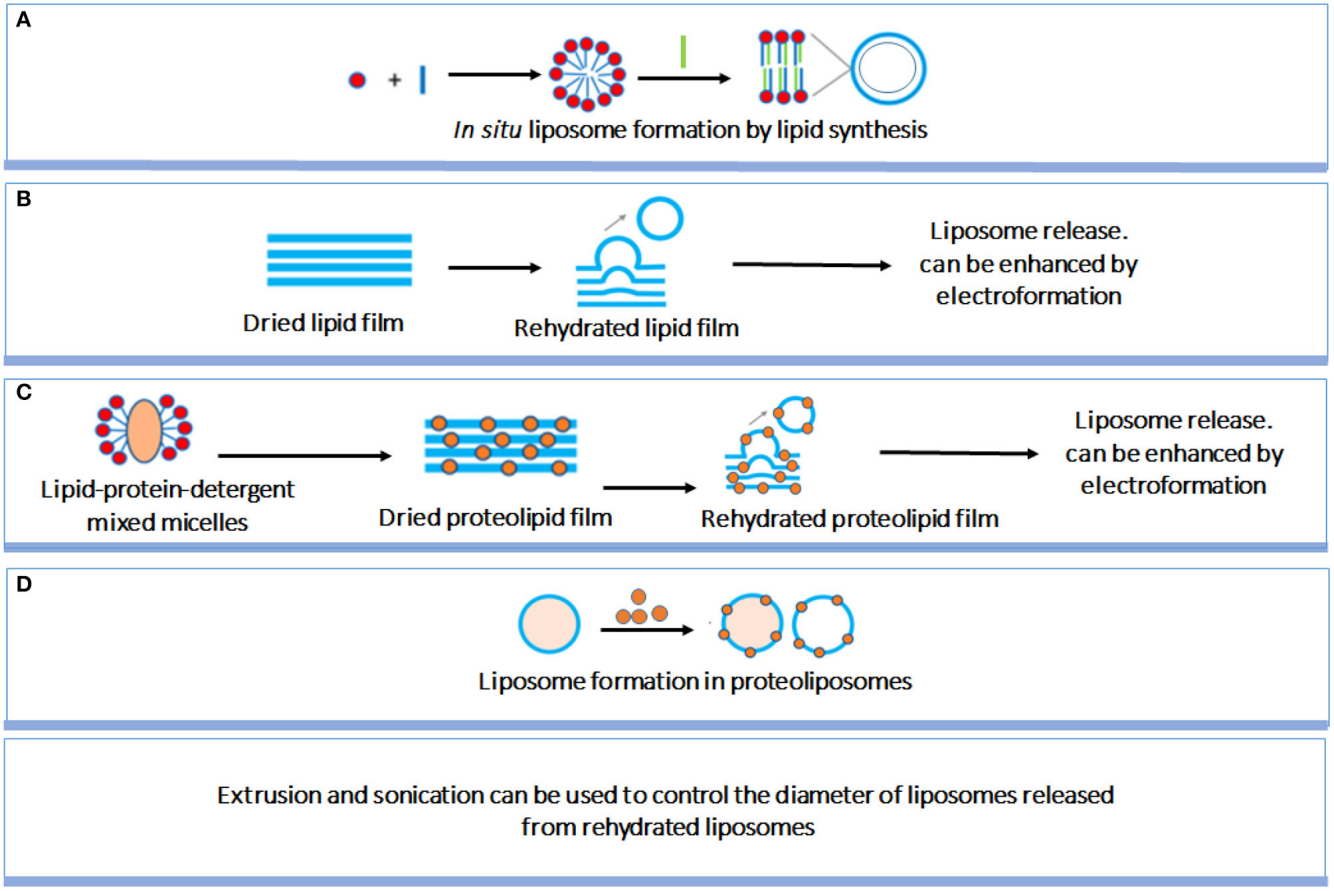
Summary of different liposome preparation methods. **(A)** *in situ* liposome formation by lipid synthesis; **(B)** Rehydration of dried lipid films yielding release of liposomes; **(C)** Similar as **(B)**, now for dried proteolipid films; **(D)** Liposome formation in proteoliposomes (Pick et al., 2018) (with permission of American Chemical Society).

Liposome preparation method is an important factor affecting the structure and size of liposomes. Although liposome preparation methods have been well-established, a short but comprehensive summary of the most used methods will be given to allow better understanding by a multi-disciplinary readership (Figure 3; Pick et al., 2018). *In situ* lipid synthesis and formation of liposomes by self-assembly into bilayered lipid structures yields liposomes of widely varying size. Liposomes can also be prepared by rehydration of dried lipid films, which spontaneously yields liposomes, with an enhanced yield when performed on conducting electrodes in the presence of an applied electric field. Liposomes size can be well-controlled by filtering, while sonication can be applied to decrease liposome size. Proteolipids can be applied in identical ways to create liposomes. Finally, large liposomes can be used to contain lipids and proteins to form proteoliposomes *in situ*, i.e., inside the larger liposomes.

## Summary of Different Types of Liposomes

Liposomes can be classified according to different criteria. Based on diameter, small (<50 nm), large (50–500 nm) and giant (>500 nm) liposomes can be distinguished (Banerjee, 2001; Morton et al., 2012). Alternatively, a classification can be made on the basis of whether a liposome possesses uni-, oligo-, or multi-lamellar bilayers (Morton et al., 2012; Manaia et al., 2017). Liposomes can consist of naturally-occurring lipids or synthetically-made lipids (sometimes called “artificial” liposomes). Accordingly, liposomes can have widely different properties and for the purpose of infection-control (i.e., interaction with negatively-charged bacterial cell surfaces; Nederberg et al., 2011; Ng et al., 2013), it is relevant to classify them into natural lipid-based, cationic, anionic, zwitterionic liposomes, and fusogenic liposomes. Diameter and diameter distribution are the most important factors for *in vivo* use of liposomes (Malekar et al., 2015) and in order to prevent rejection by the reticulo-endothelial system (Wang et al., 2019) and allow penetration through water channels (Greiner et al., 2005) in infectious biofilms, liposomes for infection-control should preferentially have diameters that maximally range up to 100–200 nm (Liu et al., 2019a). Therefore, we will now confine this review to smaller liposomes with diameters of maximally 200 nm and briefly summarize the physico-chemistry underlying these liposomes.

### Natural Lipid-Based Liposomes

**Figure 4:**
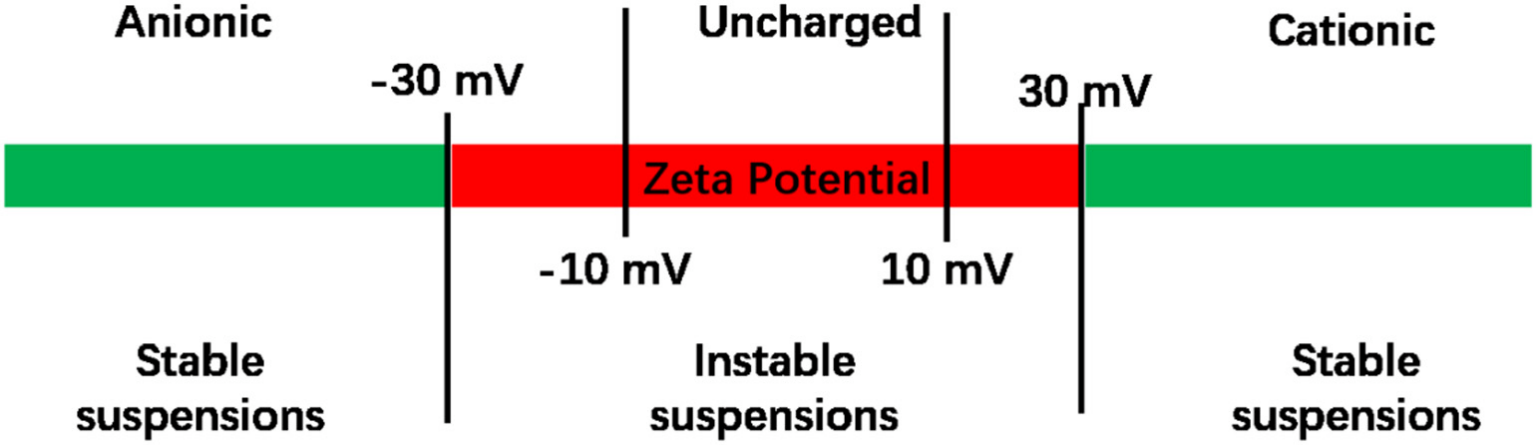
Zeta potentials of liposomes. Liposome suspensions are considered to be unstable when their zeta potential is between −30 and +30 mV (Manaia et al., 2017). Zeta potentials between −10 and +10 mV are considered to represent uncharged liposomes.

**Figure 5:**
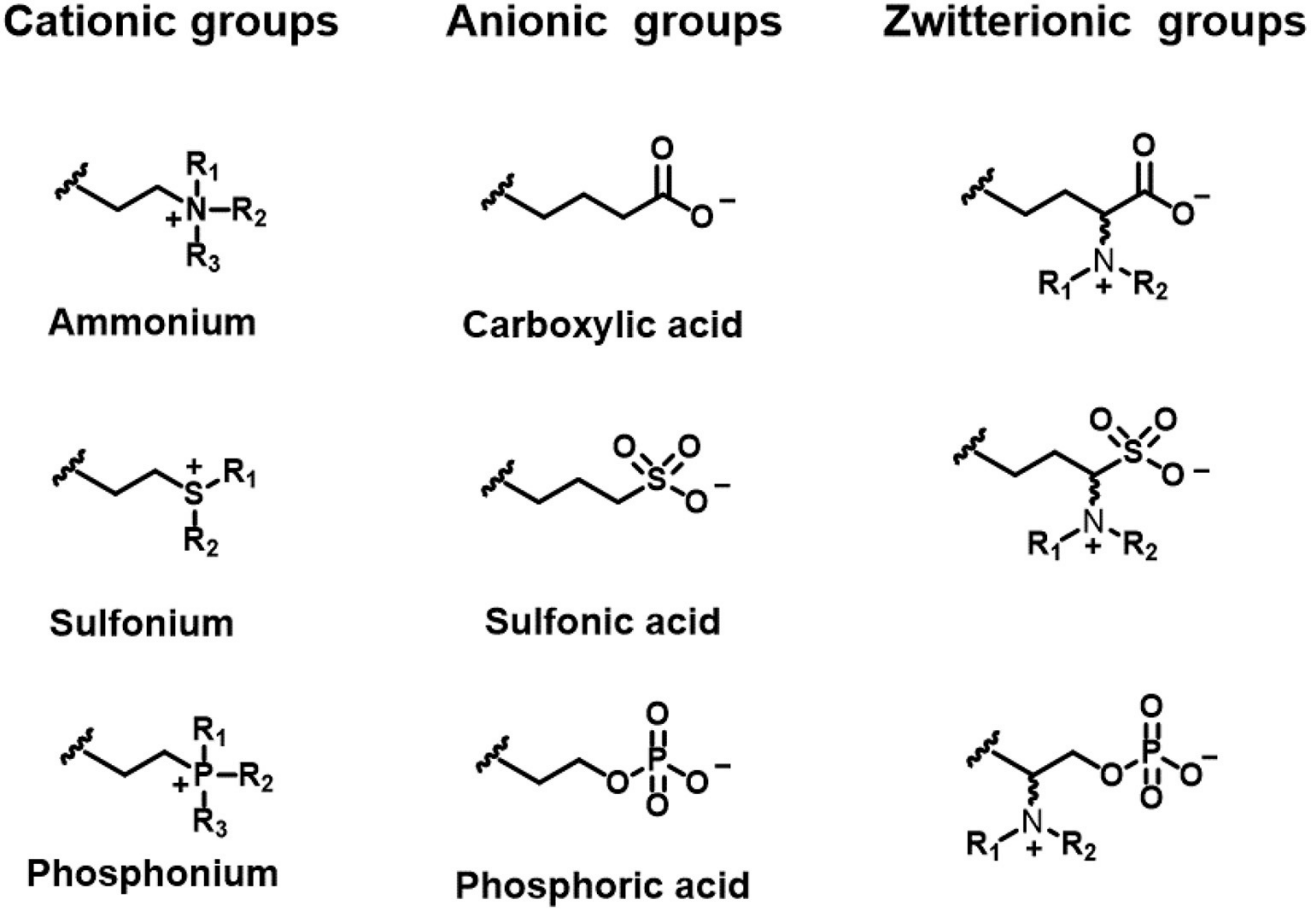
Functional groups of lipids to create differently charged liposomes.

Natural liposomes are composed of naturally-occurring phospholipids, such as phosphatidylcholine, phosphatidylserine, soybean lecithin, or egg yolk lecithin, sometimes complemented with other lipids. Natural lipids contain a polar, hydrophilic head, and several hydrophobic lipid chains. Since the hydrophilic head of natural phospholipids is electrically neutral (Smith et al., 2017), the surface potential of lipids is electrically neutral, corresponding in general with zeta potentials between −10 and +10 mV (Smith et al., 2017; Figure 4). Liposomes in suspension require zeta potentials more negative than −30 mV or more positive than +30 mV in order to experience sufficient electrostatic double-layer repulsion to create stable suspensions. Given the importance of zeta potentials for the stability of liposome suspensions and interaction with their environment, including proteins or bacteria, liposomes have been equipped with several cationic and anionic functionalities to adjust their surface charge (see also Figure 5; Kamaly et al., 2012). In addition to their stability in suspension, also the stability of the lipid bilayer in a liposome sometimes needs enforcement, such as when highly charged lipids are used (Kaszuba et al., 2010) or due to oxidation of the membrane lipids. Oxidation induced instability of liposomes can be prevented by adding reductants to the membrane lipids (Khan et al., 1990).

### Cationic Liposomes

**Figure 6:**
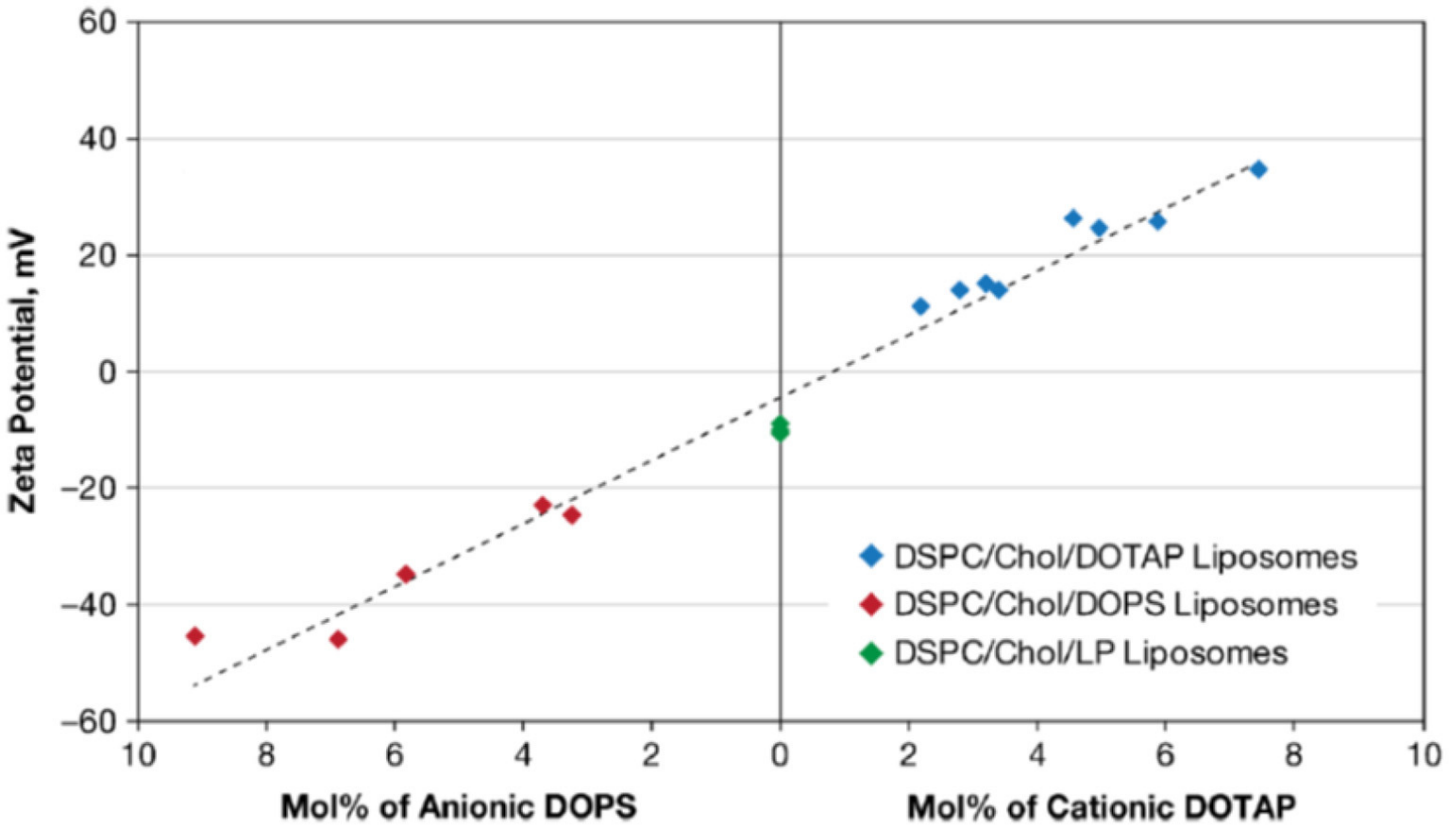
Zeta potentials in 0.01 mol/L NaCl (pH 7.4–7.7) of cholesterol (Chol), 1,2-distearoyl*sn*-glycero-3-phosphocholine (DSPC) liposomes. Liposomes were made positively-charged with varying mol% of DOTAP (1,2-dioleoyl-3-trimethylammonium-propane) or negatively-charged with DOPS (1,2-dioleoyl-sn-glycero-3-phospho-L-serine). Liposomes indicated as DSPC/Chol/LP liposomes were prepared with lipid-PEG (poly-ethylene glycol) added (Smith et al., 2017) (with permission of Springer).

Cationic liposomes can be made using natural or synthetic lipids with cationic functionalities, such as ammonium (Jacobs et al., 1916; Gottenbos et al., 2001; Lu et al., 2007), sulfonium (Ghattas and Leroux, 2009), or phosphonium ions (Popa et al., 2003; Chang et al., 2010; Figure 5). As an example, Figure 6 presents the zeta potentials of cholesterol DSPC liposomes made positively-charged through DOPA, containing positively-charged ammonium groups. Within the range of DOPA concentrations applied, zeta potentials remained below the critical limit of +30 mV required for stable suspensions and accordingly these liposome suspensions were mentioned to aggregate within 24 h of processing. Interestingly, addition of 1.6 mol% lipid-PEG yielded a zeta potential of nearly zero. Yet, lipid-PEG containing liposome suspensions were described to be stable and stealth (Kataria et al., 2011), presumably due to steric stabilization and repulsion. Cationic liposomes have been suggested as a drug-releasing coating of natural surfaces, such as skin-associated bacteria (Sanderson and Jones, 1996) or teeth (Nguyen et al., 2013), both bearing a negative charge.

**Figure 7:**
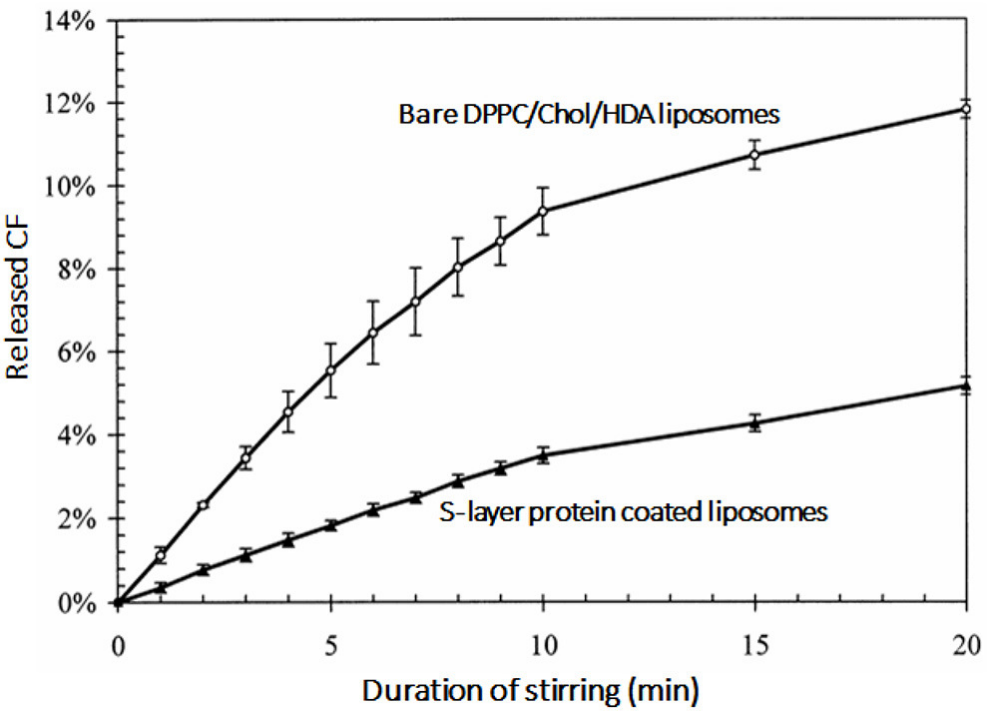
Release of fluorescent carboxyfluoresceine (CF) as an indication of the lipid bilayer stability of dipalmitoylphosphatidylcholine (DPPC), cholesterol and hexadecylamine (HDA) liposomes as a function of stirring time in the absence and presence of a bacterial S-layer coating on the liposomes (Mader et al., 1999) (with permission of Elsevier).

Instability of the liposomal bilayer structure in drug-loaded liposomes can result in inadvertent drug leakage (Drulis-Kawa and Dorotkiewicz-Jach, 2010). The stability of the lipid bilayer of cationic liposomes can be increased by coating with bacterial S-layer proteins. Zeta potentials of cationic liposomes composed of dipalmitoylphosphatidylcholine (DPPC), cholesterol and hexadecylamine [HDA: (+29.1 mV)] became negatively-charged (−27.1 mV) upon coating with S-layer proteins, which increased their stability against mechanical challenges (Figure 7; Mader et al., 1999).

### Anionic Liposomes

Anionic liposomes bear negatively-charged functional groups (Figure 5), such as carboxylic (Cheow et al., 2011), phosphoric or sulfonic acid (Derbali et al., 2019; Zhang and Lemay, 2019). Cholesterol-DSPC liposomes could be made positively-charged using DOTAP, but using DOPS, negative charge could be conveyed to these liposomes in a concentration dependent fashion (Figure 6; Smith et al., 2017). As a main advantage of anionic liposomes, opposite to cationic liposomes, anionic liposomes can more effectively encapsulate positively charged antimicrobials (Messiaen et al., 2013) and prolong their release time (Kaszuba et al., 1995; Robinson et al., 1998, 2000; Tang et al., 2009). Anionic liposomes composed of DPPG and DOPC could be loaded with eight-fold higher amounts of antibiotic than uncharged, natural-lipid based liposomes (Table 2; Messiaen et al., 2013).

**Table 2:** Increased loading of an antibiotic in anionic liposomes.

**Liposome type**	**Zeta potential (mV)**	**Tobramycin concentration (a.u.)**
DPPC/Chol	−0.5	100
DOPC/DPPG	−22.3	800

*Tobramycin-loading in anionic liposomes is eight times higher than in natural neutral liposomes due to the electrostatic interaction between negatively charged lipids and tobramycin. Data taken from Messiaen et al. (2013)*.

### Zwitterionic Liposomes

**Figure 8:**
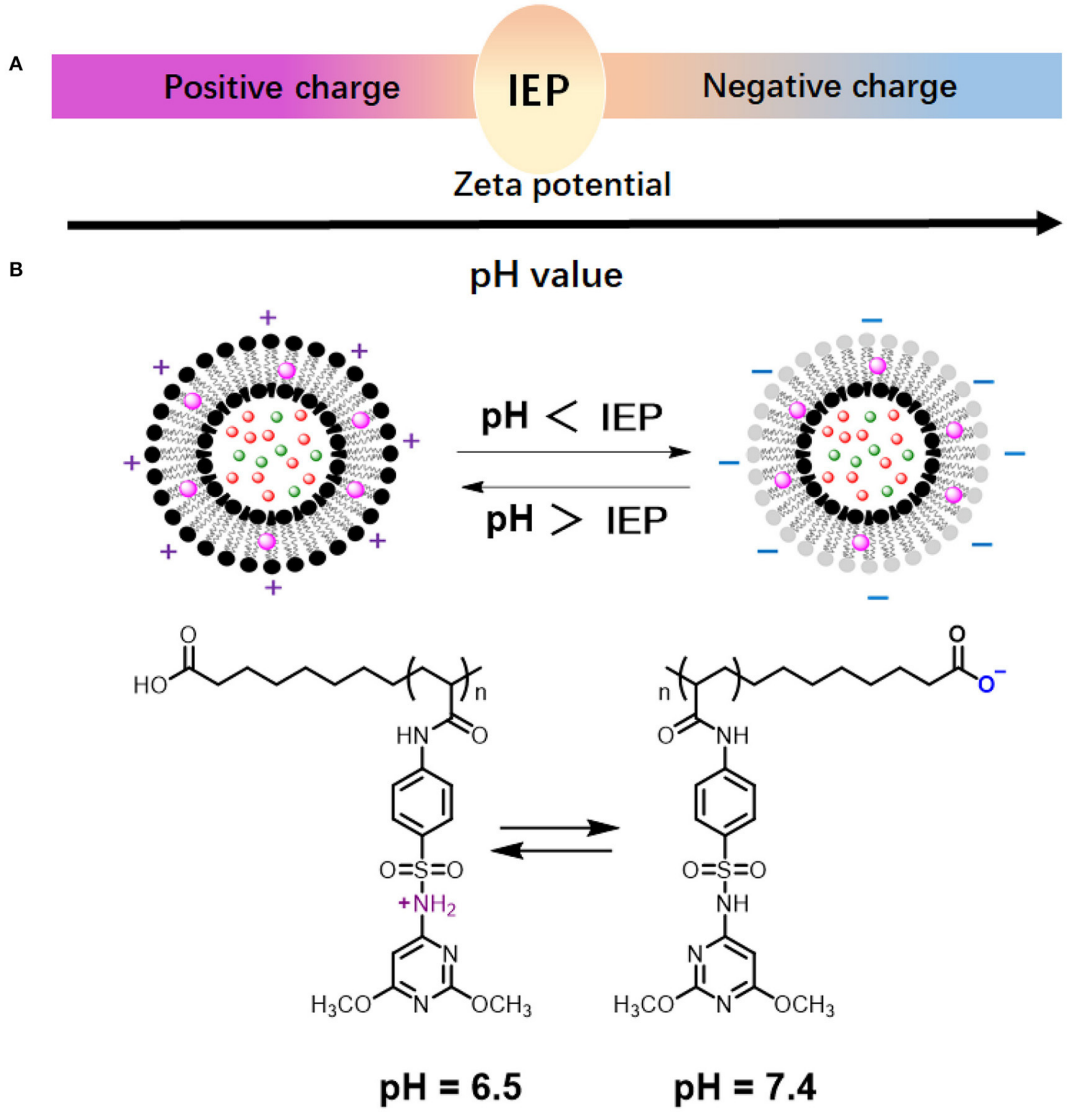
pH-dependent behavior of zwitterionic lipids and liposomes. **(A)** Zwitterionic liposomes reverse their charge from cationic to anionic when suspension pH increases from below to above the Iso-Electric Point (IEP) of the constituting lipids or vice versa. **(B)** Charge reversal of poly(methacryloyl sulfadimethoxine) (PSD) liposomes (Chen et al., 2018) (with permission of Elsevier).

Whereas, cationic and anionic liposomes usually demonstrate pH-dependent zeta potentials, they do not show complete charge reversal from being positively to negatively charged. Zwitterionic lipids have both acidic and alkaline functional groups (Figure 5; Hu et al., 2019; Makhathini et al., 2019) that allow full charge reversal below and above their iso-electric point (Figure 8A; Vila-Caballer et al., 2016; Liu et al., 2018). This feature allows the fabrication of liposomes that are negatively-charged under physiological pH conditions and become positively-charged under more acidic conditions, such as poly(methacryloyl sulfadimethoxine) (PSD) liposomes (Figure 8B; Couffin-Hoarau and Leroux, 2004; Ghattas and Leroux, 2009; Lu et al., 2018). Negative charge at physiological pH values aids transport of liposomes through the blood circulation without major interaction with other negatively-charged blood components (Hamal et al., 2019), while adaptation of a positive charge inside the acidic environment of a biofilm facilitates better interaction with negatively-charged bacteria (Robinson et al., 2001; Nederberg et al., 2011; Ng et al., 2013) in the biofilm.

### Fusogenic Liposomes

**Figure 9:**
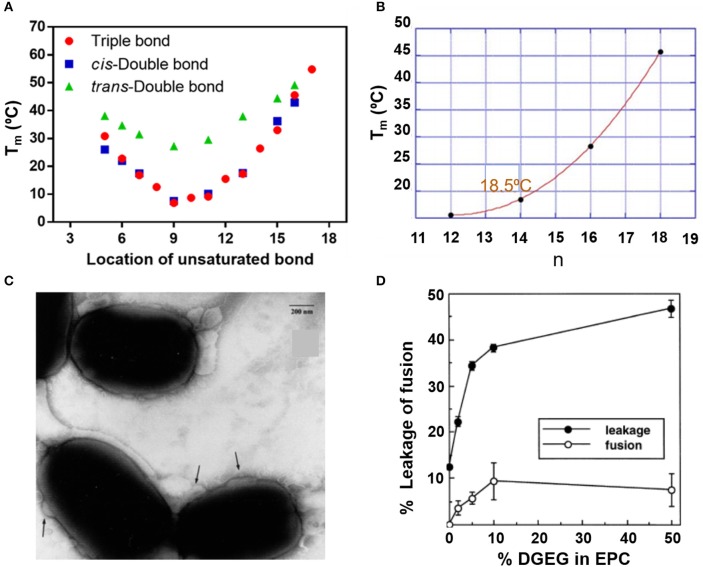
Fluidity of liposomes in relation with their lipid structure. Melting temperature T_m_ of lipids as an indication of fluidity. **(A)** Melting temperature as a function of unsaturated bond location in (f sn-1 saturated/sn-2 monosaturated phosphatidylcholine) (Nagahama et al., 2007). **(B)** Melting temperature of 5.0 mM dialkyldimethylammonium bromide in water as a function of the number (n) of carbon atoms in the alkyl chains (Feitosa et al., 2006) (with permission of Elsevier). **(C)** Transmission electron micrographs of the fusion (indicated by the arrows) of fusogenic, DOPE-DPPC-cholesterol hemisuccinate liposomes with *E. coli*. Bar marker equals 200 nm (Nicolosi et al., 2010) (with permission of Elsevier). **(D)** The % fused lipsosomes and % release of fluorescent carboxyfluorescein as a function of the % digalactosyldiacylglycerol (DGDG) in egg phosphatidylcholine (EPC) liposomes (Hincha et al., 1998) (with permission of Elsevier).

The fusogenicity of liposomes with cellular membranes is a most distinguishing feature of liposomes and is related with the fluidity of the lipid bilayer. Generally, lower melting temperatures of the lipids imply higher fluidity of the liposome membrane and therewith a greater fusogenicity (Zora and Željka, 2016). Figure 9 summarizes the relation between melting temperatures and structure/composition of lipids. Both location of unsaturated bonds (Figure 9A; Nagahama et al., 2007) and alkyl chain length (Figure 9B) influence lipid melting temperatures (Feitosa et al., 2006) and therewith the fusogenicity of liposomes. Cholesterol hemisuccinate for instance, combined with dioleoylphosphatidylethanolamine (DOPE) and dipalmitoylphosphatidylcholine (DPPC) in a 4:2:4 molar ratio yielded highly fusogenic liposomes (Figure 9C). Increasing fusogenicity however, may go at the expense of the stability of the lipid bilayer constituting the membrane and liposomes with increased fusogenicity are more prone to bilayer membrane instability, rupture, and inadvertent cargo release (Marier et al., 2002; Li et al., 2013; Figure 9D).

## Application of Antimicrobial-Loaded Liposomes Toward Infectious Biofilms

The problems to be overcome for the successful treatment of infectious biofilms in the human body are many-fold and some of them have persisted for centuries. Rather than aiming for a comprehensive overview of all studies attempting to apply liposomal antimicrobial-loaded nanocarriers for infection-control, we first present a brief overview of the problems encountered in the treatment of infectious biofilms using antimicrobials. Next, it will be addressed which problems can probably be successfully addressed using liposomal antimicrobial-loaded nanocarriers, and the steps that need to be taken for successful downward clinical translation.

### Traditional Problems in Antimicrobial Treatment of Infectious Biofilms

**Figure 10:**
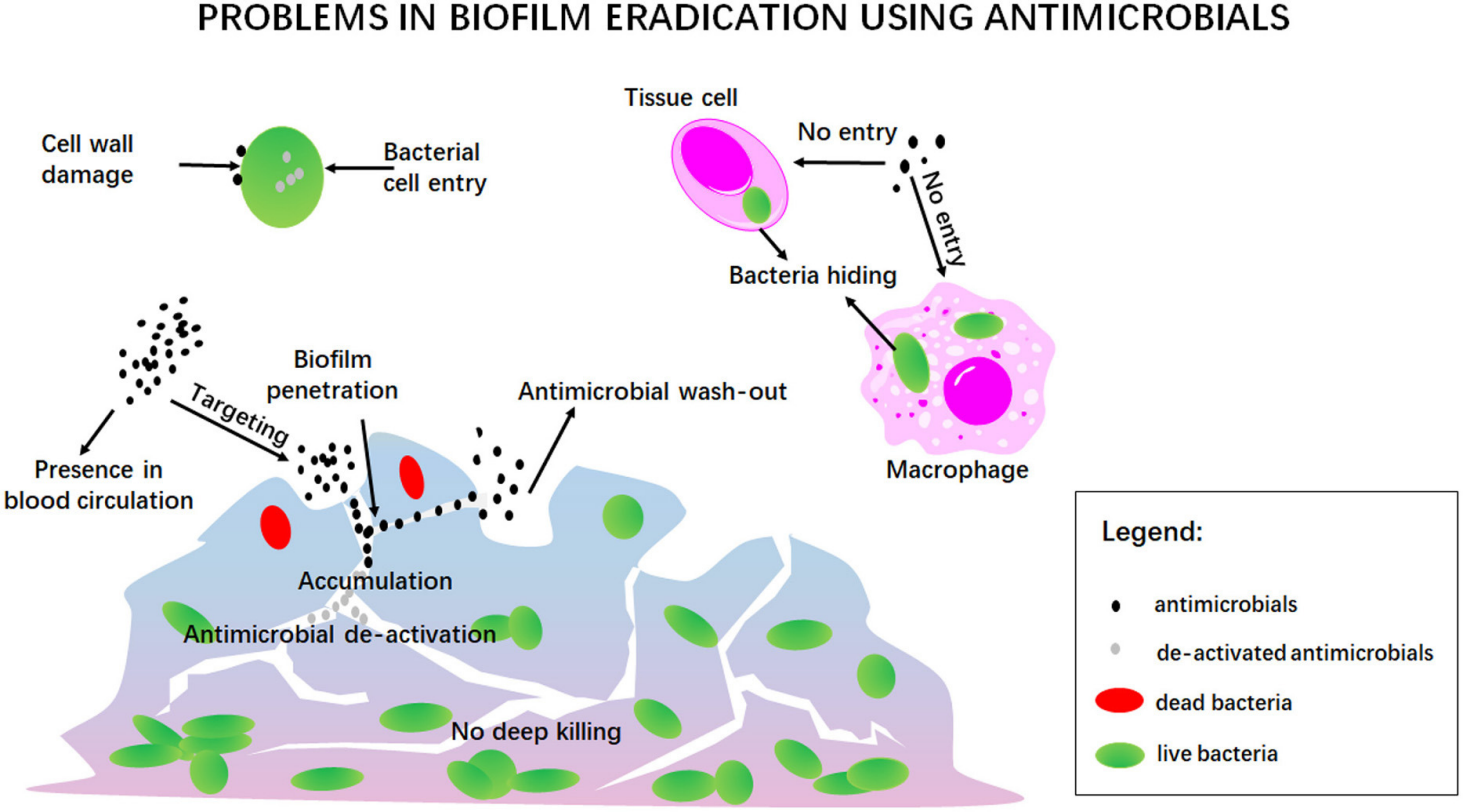
A summary of the traditional problems involved in antimicrobial treatment of infectious biofilms.

Eradication of infectious biofilms is a highly complicated process for which there is no adequate treatment available ever since Van Leeuwenhoek noticed that the vinegar which he used to clean his teeth from oral biofilm killed only bacteria residing at the outside of the biofilm, but left the ones in the depth of a biofilm alive (Van Leewenhoek, 1684). One of the current struggles indeed, still is the penetration, accumulation and killing of antimicrobials over the entire thickness of an infectious biofilm, as noticed by Van Leeuwenhoek over three centuries ago (Figure 10). This includes prevention of wash-out of an antimicrobial in the dynamic environment of the human body. In addition, antimicrobials may be enzymatically de-activated underway to a biofilm in the blood circulation or once inside a biofilm (Albayaty et al., 2018). Taken together, these factors make bacterial killing into the depth of a biofilm impossible (Sutherland, 2001), contributing to recurrence of infection after treatment (Wolfmeier et al., 2018).

Penetration and accumulation can only occur once the antimicrobial has “found its way,” often from within the blood circulation, to the infectious biofilm. Since it may be undesirable to have high concentrations of an antimicrobial circulating through the body due to potential collateral tissue damage, self-targeting carriers are under design that can find their way at low blood concentrations to accumulate in sufficiently high amounts in an infectious biofilm (Forier et al., 2014). Once accumulated inside a biofilm, the antimicrobial should perform its antimicrobial action, which can either be based on generating cell wall damage, or entry into a bacterium to interfere with vital metabolic processes. Both can be difficult, especially since bacteria have developed a large array of protective mechanisms, that we summarize under the common denominator of antimicrobial resistance (Kumar et al., 2016). Adding to this, is the problem of bacteria seeking shelter against antimicrobials in mammalian cells (Mantovani et al., 2011), in which many antimicrobials cannot enter. Bacteria have even been found sheltering in macrophages intended by nature to kill them, de-activating macrophageal killing mechanisms (Knodler et al., 2001).

There are no antimicrobials or antimicrobial carriers that solve all the issues summarized above (see also Figure 10). Liposomal nanocarriers constitute no exception to this. Yet, liposomes possess a number of unique qualities, like stealth properties, protection of encapsulated antimicrobials against de-activation and entry in tissue cells and bacteria, as will be summarized below.

### Solutions to Traditional Problems in Antimicrobial Treatment of Infectious Biofilms Offered by Liposomal Antimicrobial Nanocarriers

Blood circulation times of liposomes have become much longer since the inclusion of lipid-PEG in the bilayer membrane. Liposomes without lipid-PEG were rapidly removed from the circulation by macrophageal uptake (Hofmann et al., 2010) but stealth (Romberg et al., 2008) liposomes containing lipid-PEG demonstrated reduced reticulo-endothelial uptake.

Generally, cationic liposomes demonstrate better interaction with negatively charged bacterial cell surfaces (Robinson et al., 2001; Nederberg et al., 2011; Ng et al., 2013). However, pH-responsive liposomes that self-target from the blood circulation toward bacteria in an infectious biofilm have not been extensively explored. Zwitterionic liposomes prepared from pH-responsive quaternary ammonium chitosan with charge reversal from −9.08 mV at pH 7.4 to +8.92 mV at pH 4.5 have been described for the treatment of periodontal infection (Zhou et al., 2018; Hu et al., 2019). However, according to Figure 4 this change does not qualify as a charge reversal as these liposomes would have to be classified as uncharged at both pH values. Moreover, periodontal application does not imply self-targeting from the blood circulation, as required for the treatment of many other, internal infections. Interestingly, these zwitterionic liposomes were highly biocompatible and disruptive to periodontal biofilm.

Many Gram-negative and Gram-positive bacterial strains, resistant to a specific antibiotic free in solution, have been demonstrated to be susceptible to these antibiotics when encapsulated in a liposomal nanocarrier (Table 3). This may arguably be considered as the biggest advantage of liposomes over other nanocarriers. Although some have suggested that this must be attributed to the protection offered by liposomal encapsulation against enzymatic de-activation (Nacucchio et al., 1985), fusogenicity (Mugabe et al., 2006; Halwani et al., 2007) of liposomes can also significantly improve the antibacterial activity of antibiotics (Beaulac et al., 1996; Sachetelli et al., 1999; Li et al., 2013). Liposomes with enhanced fusogenicity possessing cholesterol hemisuccinate (Nicolosi et al., 2010) loaded with vancomycin for instance, had much lower minimal inhibitory concentrations (MIC) than vancomycin free in solution against a variety of Gram-negative bacterial strains, that would be considered vancomycin-resistant based on their MIC (see also Table 3). Also fusogenic liposomes composed of dipalmitoylphosphatidylcholine (DPPC) and dimiristoylphosphatidylglycerol (DMPG) in a ratio of 18:1 (w/w) loaded with tobramycin eradicated a mucoid chronic, pulmonary *Pseudomonas aeruginosa* infection, whereas tobramycin free in solution was not effective (Beaulac et al., 1996, 1998).

**Table 3:** Minimal inhibitory concentrations of different bacterial strains against antibiotic-loaded liposomes.

**Strain**	**MIC against free antibiotics (mg/L)**	**MIC against liposomal encapsulated antibiotics (mg/L)**	**References**
**Vancomycin**			
*E. coli*	512	6–25	Nicolosi et al., 2010
	512	10.5	
*Klebsiella*	512	25–50	
*P. aeruginosa*	512	50	
	512	83.7	
*Acinetobacter baumanii*	512	6–125	
*S. aureus (MRSA)*	1	0.5	Bhise et al., 2018
**Amikacin**			
*P. aeruginosa*	8	4	Mugabe et al., 2006
	16	4	
	252	8	
	4	2	
	512	8	
*B. cenocepacia*	256	8	Halwani et al., 2007
	256	32	
	128	16	
	>512	8	
	4	1	
**Gentamicin**			
*P. aeruginosa*	4	2	Mugabe et al., 2006
	16	2	
	32	4	
	32	0.5	
	256	8	
*B. cenocepacia*	>512	32	Halwani et al., 2007
	256	64	
	256	16	
	>512	32	
	1	0.25	
**Tobramycin**			
*P. aeruginosa*	2	1	Mugabe et al., 2006
	4	4	
	64	2	
	1	0.5	
	1024	8	
*B. cenocepacia*	512	8	Halwani et al., 2007
	>512	64	
	128	16	
	>512	16	
	1	0.25	
**Piperacillin**			
*S. aureus*	64	32	Nacucchio et al., 1985
**Cefepime**			
*P. aeruginosa*	8	4	Torres et al., 2012
**Ceftazidime**			
*P. aeruginosa*	8	4	Torres et al., 2012
**Levofloxacin**			
*P. aeruginosa*	0.5	0.5	Derbali et al., 2019
**Voriconazole**			
*Aspergillus* sp.	0.5	0.5	Veloso et al., 2018
	0.25	0.25	
	0.5	0.25	
	1	0.5	
*Candida* sp.	0.03	0.03	
	0.06	0.06	
	0.03	0.03	
	0.03	0.03	
**Meropenem**			
*P. aeruginosa*	125	1.5	Zahra et al., 2017
	62.5	6.25	
	62.5	6.25	
	125	50	
	250	100	
	25	6.25	
	250	100	
	62.5	6.25	
	250	50	
**Clarithromycin**			
*S. aureus* ATCC29213	0.25	0.25	Meng et al., 2016
*S. aureus MRSA*	64	16	
*P. aeruginosa*	>256	8–64	Alhajlan et al., 2013
	256	8–64	
	256	8–64	
	256	8–6,432; 64; 8	
	>256	8–64	
	>256	8–64	
	>256	8–64	
	>256	8–64	
	256	8–64	
**Azithromycin**			
*P. aeruginosa*	128	16	Solleti et al., 2015
	64	8	
	512	32	
	128	16	
	256	32	
	512	32	
	512	128	
	256	32	
	512	64	
	256	16	

*Multiple MIC values for the same strain, antibiotic and reference, refer to different isolates of the same strain or different liposomes in the same reference*.

## Perspectives of Lipid-Based Antimicrobial Nanocarriers for Treating Bacterial Biofilm Infection

Protection of antibiotics against enzymatic de-activation and fusogenicity to enhance antibiotic efficacy, constitute unique advantages of liposomal antimicrobial nanocarriers that justify further research. Challenges in the ongoing development of liposomal antimicrobial nanocarriers include the realization of biofilm targeting from the blood circulation, penetration, and accumulation over the entire thickness of an infectious biofilm, associated with deep killing in the biofilm. Deep killing is necessary in order to prevent recurrence of infection, one of the troublesome features of clinical infection treatment. In this respect, it is also worthwhile to investigate whether liposomal antimicrobial nanocarriers can be designed that aid in the killing of bacteria seeking shelter in mammalian cells, impenetrable to many antimicrobials.

Downward clinical translation of liposomal drug nanocarriers has long been hampered for difficulties in large-scale production and storage. However, ethanol injection, membrane dispersion, and Shirasu porous glass membranes have enabled large-scale production of liposomes (Laouini et al., 2012). Equally, liposome storage problems are on their way to be solved. For commercial liposome products, storage in the fluid form is preferred since lyophilization and subsequent rehydration may lead to size changes and cargo leakage (Stark et al., 2010). Addition of stabilizers such as 2-morpholinoethansulfonic acid yielded low phospholipid degradation in liposomes after 12 months storage at 2–8°C (Doi et al., 2019).

Owing to these developments, liposomes are nowadays an FDA approved form of drug delivery and liposome encapsulated tobramycin, marketed under the name Fluidosomes™ is clinically applied for the treatment of chronic pulmonary infections in cystic fibrosis patients. A phase II clinical study is ongoing in Europe (Zora and Željka, 2016).

In conclusion, the challenges to further develop liposomes as a novel infection-control strategy supplementing antibiotic treatment are highly worthwhile to pursue.

## Author Contributions

All authors listed have made a substantial, direct and intellectual contribution to the work, and approved it for publication.

### Conflict of Interest

HB is also director of a consulting company, SASA BV. The remaining authors declare no conflicts of interest with respect to authorship and/or publication of this article. Opinions and assertions contained herein are those of the authors and are not construed as necessarily representing views of their respective employers.

## Abbreviations

CF: carboxyfluoresceine
Chol: cholesterol
DGDG: digalactosyldiacylglycerol
DMPC: dimyristoyl phosphatidylcholine
DMPG: dimyristoyl phosphatidylglycerol
DOPA: 3,4-dihydroxyphenylalanine
DOPC: 1,2-dioleoyl-sn-glycero-3-phosphocholine
DOPE: 1,2-dioleoyl-3-trimethylammonium-propane
DOPS: 1,2-dioleoyl-sn-glycero-3-phospho-L-serine
DOTAP: 1,2-dioleoyl-3-trimethylammonium-propane
DPPC: dipalmitoyl phosphatidylcholine
DPPG: dipalmitoylphosphatidylglycerol
DSPC: 1,2-distearoylsn-glycero-3-phosphocholine
DSPE: distearoyl phosphoethanolamine
EPC: egg phosphatidylcholine
EPS: extracellular polymeric substances
FDA: food and drug administration
HAD: hexadecylamine
IEP: iso-electric point
LP: lipid-PEG
MOFs: metal organic framework
MIC: minimal inhibitory concentration
MRSA: methicillin-resistant *Staphylococcus aureus*
NPs: nanoparticles
PAE: poly(β-amino ester)
PEG: polyethylene glycol
PSD: poly(methacryloyl sulfadimethoxine).
